# Plasma Metabolite Profiles Between In-Center Daytime Extended-Hours and Conventional Hemodialysis

**DOI:** 10.34067/KID.0000000675

**Published:** 2024-12-09

**Authors:** Norito Takami, Masaki Okazaki, Takaya Ozeki, Takahiro Imaizumi, Nobuhiro Nishibori, Shimon Kurasawa, Manabu Hishida, Shin'ichi Akiyama, Rintaro Saito, Akiyoshi Hirayama, Hirotake Kasuga, Fumika Kaneda, Shoichi Maruyama

**Affiliations:** 1 Department of Nephrology, Nagoya University Graduate School of Medicine, Nagoya, Japan; 2 Department of Clinical Research Education, Nagoya University Graduate School of Medicine, Nagoya, Japan; 3 Department of Advanced Medicine, Nagoya University Hospital, Nagoya, Japan; 4 Department of Nephrology, Kaikoukai Josai Hospital, Nagoya, Japan; 5 Department of Nephrology, Institute for Advanced Biosciences, Keio University, Yamagata, Japan; 6 Department of Nephrology, Nagoya Kyoritsu Hospital, Nagoya, Japan; 7 Kamome Clinic, Hitachi, Japan

**Keywords:** chronic hemodialysis, dialysis, metabolomics

## Abstract

**Key Points:**

- Significant differences in 39 plasma metabolites were observed between patients on extended-hours hemodialysis and those on conventional hemodialysis.
- Extended-hours hemodialysis had a lower lactate-to-pyruvate ratio and higher branched-chain amino acids than conventional hemodialysis.
- Extended-hours hemodialysis may have favorable metabolic and nutritional benefits for patients undergoing maintenance hemodialysis.

**Background:**

Protein–energy wasting, characterized by disordered body protein catabolism resulting from metabolic and nutritional derangements, is associated with adverse clinical outcomes in patients undergoing hemodialysis. Extended-hours hemodialysis (≥6 hours per treatment session) offers both enhanced removal of uremic solutes and better fluid management, generally allowing more liberalized dietary protein and calorie intake. The aim of this study was to evaluate the difference in plasma metabolite profiles among patients receiving in-center daytime extended-hours hemodialysis and those receiving conventional hemodialysis.

**Methods:**

Predialysis plasma samples were obtained from 188 patients on extended-hours hemodialysis (21.9 h/wk) and 286 patients on conventional hemodialysis (12.1 h/wk) in Japan in 2020 using capillary electrophoresis-mass spectrometry. Group differences were compared for 117 metabolites using Wilcoxon rank-sum tests with multiple comparisons and partial least squares discriminant analysis. In addition, propensity score–adjusted multiple regression analyses were performed to evaluate group differences for known uremic toxins, branched-chain amino acids, and lactate-to-pyruvate ratio (a possible surrogate marker of mitochondrial dysfunction).

**Results:**

Significant differences were observed in 39 metabolites, largely consistent with the high variable importance for prediction in partial least squares discriminant analysis. Among known uremic toxins, uridine and hypoxanthine levels were significantly higher in the conventional hemodialysis group than in the extended-hours hemodialysis group, whereas trimethylamine *N*-oxide levels were higher in the extended-hours hemodialysis group than in the conventional hemodialysis group. Patients on extended-hours hemodialysis had higher levels of all branched-chain amino acids and a lower lactate-to-pyruvate ratio than those on conventional hemodialysis (significant difference of −8.6 [95% confidence interval, −9.8 to −7.4]).

**Conclusions:**

Extended-hours hemodialysis was associated with a more favorable plasma metabolic and amino acid profile; however, concentrations of most uremic toxins did not significantly differ from those of conventional hemodialysis.

## Visual Abstract

**Figure:**
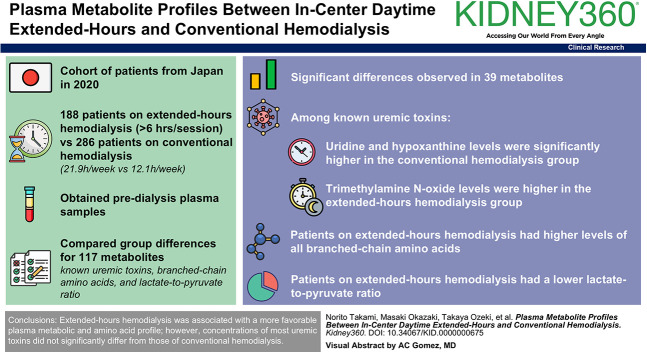

## Introduction

Protein–energy wasting is a state of nutritional and metabolic derangements in patients with CKD characterized by loss of systemic body protein and energy stores.^1–3^ It is observed in 28%–54% of patients on maintenance dialysis and is associated with unfavorable clinical phenotypes, including impaired physical activity, muscle wasting, frailty, and ultimately high mortality.^1,2,4^ Tackling protein–energy wasting in patients on hemodialysis requires an integrative approach aimed at addressing both the negative protein balance and uremic milieu, which negatively affects body protein anabolism.^3,5^ The influx of amino acids from dietary protein intake is certainly a necessary raw material for protein synthesis in the body, but the dilemma is that the metabolic products of dietary protein itself are eventually turned into uremic toxins.^6,7^ Accumulation of uremic retention solutes can contribute to the hypercatabolic state encompassed by protein–energy wasting by altering physiological protein metabolism.^2,3,8^

Extended-hours hemodialysis (usually 3–4 times weekly, 6–8 h/session) can offer both enhanced removal of uremic retention solutes and better fluid management, which generally allows a more liberalized dietary intake. Several large-scale cohort studies have shown that extended-hours hemodialysis is associated with better removal of phosphate and improved nutritional indicators, hypertension, erythropoiesis-stimulating agent resistance, and mortality.^9–11^ An intensive hemodialysis regimen may dilute and/or lower the concentration of potentially toxic retained uremic solutes; however, previous randomized controlled studies of Frequent Hemodialysis Network trials did not present the apparent nutritional benefits of frequent nocturnal hemodialysis (six times weekly, 6–8 h/session) treatment at home.^12,13^ A possible explanation for this finding is that the participants in the Frequent Hemodialysis Network Nocturnal Trial were largely incident patients who were new to dialysis with substantial baseline urine volumes and residual kidney function, which clears numerous uremic retention solutes from plasma of patients with kidney disease.^12,14,15^

Untargeted metabolomics is the study of comprehensive identification that aims to simultaneously measure as many metabolites as possible from biological samples.^16^ A previous cohort study in Canada using this metabolomic approach reported minimal changes in plasma metabolites, such as uremic toxins, despite a considerable increase in treatment duration 1 year after transitioning from conventional hemodialysis to extended-hours nocturnal hemodialysis.^17^ However, the patients undergoing nocturnal hemodialysis administered overnight at a hospital in this prior study were relatively young and nourished, with a mean age of 58 years and a mean body mass index (BMI) of 28.4 kg/m^2^. Thus, the metabolic profile of older individuals, who are presumed to be more susceptible to protein–energy wasting, remains unclear. We hypothesize that extended-hours hemodialysis is associated with a favorable plasma metabolite profile, reflecting improved metabolic and nutritional status. The aim of this study was to comprehensively evaluate the plasma metabolites using the largest multicenter cohort of patients undergoing in-center extended-hours hemodialysis in Japan. We compared uremic retention solutes, lactate-to-pyruvate ratio (a possible surrogate marker of mitochondrial dysfunction^18–20^), and amino acid status between the extended-hours hemodialysis and conventional hemodialysis groups.

## Methods

### Study Population

This was a multicenter study using predialysis blood samples collected from January to March 2020 at two dialysis facilities providing in-center daytime extended-hours hemodialysis (nocturnal hemodialysis, which treats patients overnight, is not available at these facilities) and one facility offering in-center conventional hemodialysis. Extended-hours hemodialysis was defined as ≥6 hours per treatment session. The two extended-hours hemodialysis centers offered intradialytic regular meals constantly during hemodialysis treatment (Supplemental Table 1), whereas the conventional hemodialysis center prohibited almost all food consumption inside the facility in consideration of the coronavirus disease 2019 pandemic. This study included patients with at least 1 month of continuous dialysis, minimizing confounding factors, such as dialysis prescription and dietary changes. We enrolled patients on daytime hemodialysis who consented to participate in this study at the three sites and obtained blood samples (*n*=483). All patients were outpatients, and no patients with acute illness were included. We excluded patients with poor specimens (*n*=8) and missing clinical information (*n*=1) and evaluated samples from patients on extended-hours hemodialysis (*n*=188) and conventional hemodialysis (*n*=286). Written informed consent was obtained from all participating patients, and ethical approval was granted by the Institutional Review Board of Nagoya University (approval no. 2014-0422).

### Metabolite Profiling and Data Collection

Blood samples were obtained at each dialysis center at the beginning of the week before the start of the dialysis session. The collected blood samples were promptly frozen at −80°C using EDTA disodium collection tubes for plasma samples and after coagulation for serum samples. Metabolomic analysis of plasma samples was performed at the Institute for Advanced Biosciences, Keio University using capillary electrophoresis time-of-flight mass spectrometry (CE-TOFMS). CE-TOFMS analysis was performed using an Agilent CE-TOFMS system (Agilent Technologies, Waldbronn, Germany). A brief description of the sample preparation and measurement process is provided in Supplemental Method 1. Detailed measurement conditions for both cationic and anionic metabolites by CE-TOFMS have been described in the literature,^21^ and the same procedure was used. Master Hands version 2.19.0.0 software,^22,23^ developed at the Institute for Advanced Biosciences, Keio University, was used for peak detection and quantification of metabolites from the raw data obtained by CE-TOFMS. Biochemical data for serum albumin, creatinine, calcium, phosphate, intact parathyroid hormone, and C-reactive protein levels were measured by LSI Med (Aichi, Japan) using serum samples obtained from the study. Serum albumin levels were measured using the modified bromocresol purple method. Serum *β*2-microglobulin data were collected from predialysis blood tests during the same month as the sample collection. Data regarding demographic characteristics, dialysis prescriptions, and hemodialysis conditions, including single-pool Kt/V_urea_ and normalized protein catabolic rate (nPCR) calculated by the method proposed by Shinzato *et al*.,^24^ were collected from electronic medical records and managed using an electronic data capture system.

### A Possible Surrogate Marker of Mitochondrial Dysfunction

As the well-known clinical feature of mitochondrial diseases is lactic acidosis, lactate, pyruvate, and amino acids are considered candidate biomarkers of mitochondrial dysfunction.^25^ Notably, the plasma lactate-to-pyruvate ratio, which reflects the cytoplasmic redox state,^26^ has been used as one of the promising biomarkers of mitochondrial dysfunction.^18–20^ Although the lactate-to-pyruvate ratio in a healthy human is approximately ten in arterial blood,^27^ the ratio increases in the presence of mitochondrial dysfunction, owing to enhanced anaerobic metabolism and facilitated conversion from pyruvate to lactate.^19,28,29^ In this study, the plasma lactate-to-pyruvate ratio was used as a surrogate marker of mitochondrial dysfunction.

### Statistical Analyses

Patient characteristics are expressed as means (SDs) or medians (interquartile ranges [IQRs]) for continuous variables and percentages (%) for categorical variables. Group comparisons were conducted using chi-squared tests for categorical variables and *t* tests or Wilcoxon rank-sum tests for continuous variables, as appropriate. The statistical analysis focused on 117 plasma metabolites with measurement values of ≥70% in either extended-hours or conventional hemodialysis (Supplemental Table 2). Wilcoxon rank-sum tests, principal component analysis (PCA), and partial least squares discriminant analysis (PLS-DA) were performed for these 117 plasma metabolites. Bonferroni correction was applied for multiple comparisons, with an adjusted significance threshold of *P* < 0.00043 (0.05/117) for Wilcoxon rank-sum tests. PCA and PLS-DA were used to evaluate the data distribution of metabolites among samples across the treatment groups. In multivariable analyses, missing value imputation was performed using the limit of detection (one fifth of the minimum positive value of each variable), and data scaling was performed by autoscaling (mean centered and divided by the SD of each variable). Score plots from the PCA and PLS-DA were visually assessed to evaluate the distinction between the extended-hours hemodialysis and conventional hemodialysis groups. PLS-DA provides variable importance for prediction (VIP) scores to identify metabolites with high variable importance in the model (VIP ≥1.0).^30^ Quantitative enrichment analysis (QEA) was performed using the Kyoto Encyclopedia of Genes and Genomes database,^31^ based on statistically significant metabolites determined by both the Wilcoxon rank-sum test and PLS-DA. The enrichment ratio was computed by dividing the number of hits within a particular metabolic pathway by the expected number of hits. PCA, PLS-DA, and QEA were performed using MetaboAnalyst 6.0 software (www.metaboanalyst.ca).^32^

Propensity scores were calculated using multiple logistic regression models to account for potential confounders. The covariates used for the calculation of propensity scores were age, sex, time since starting dialysis, BMI, diabetes mellitus, hypertension, coronary artery, peripheral artery, and cerebrovascular diseases. Subsequently, we used an inverse probability of treatment weighting (IPTW) for multiple regression analysis to evaluate group differences for 14 uremic toxins, lactate-to-pyruvate ratio, and branched-chain amino acids (BCAAs). The weighting coefficients were 1/propensity score (PS) for patients on extended-hours hemodialysis and 1/(1−PS) for patients on conventional hemodialysis. The balance of covariates between groups was assessed by using standardized differences.^33^ Doubly robust methods^34^ were also used in the multiple regression analysis, incorporating PS and PS model variables plus medications data (*i.e*., use of phosphate binders, active vitamin D, and calcimimetics). Analysis for uremic toxins applied Bonferroni adjustment for multiple comparisons with an adjusted significance threshold of *P* < 0.0036 (0.05/14). The subgroup analysis for the lactate-to-pyruvate ratio considered the groups of age (<70 and ≥70 years), sex, time since starting dialysis (<1, 1–9.9, and ≥10 years), BMI (<18.5, 18.5–24.9, and ≥25 kg/m^2^), and diabetes mellitus. In this subgroup analysis, the categorical variables associated with each subgroup were excluded from the model, and the interaction terms were evaluated using Wald test. All statistical analyses, except PCA, PLS-DA, and QEA, were performed using Stata SE version 17.0 (StataCorp, College Station, TX).

### Clinical Relevance

We evaluated the overall survival and longitudinal changes in BMI as a nutritional indicator over a 4-year period from the time of sample collection to assess the clinical features between the two treatment groups, using data obtained from the electronic medical records of the participating facilities. We presented Kaplan–Meier survival curves and estimated BMI trajectories, both stratified by dialysis methods. The BMI trajectories were estimated using a linear mixed-effects model adjusted for baseline age, sex, BMI, diabetes mellitus, and time since starting dialysis. This model included random intercepts and slopes for each individual and accounted for within-patient correlation using an unstructured variance–covariance matrix. Differences in BMI trajectories between the groups were evaluated using the Wald test for the interaction term between dialysis methods and time.

## Results

The demographic and clinical characteristics are summarized in Table 1. The mean ages were 66.1 years for the extended-hours hemodialysis group and 68.6 years for the conventional hemodialysis group. In the extended-hours hemodialysis group, patients had a longer time since starting dialysis (9.1 [IQR, 3.4–15.8] versus 6.0 [2.5–13.3] years) and higher BMI (22.8 [IQR, 21.0–26.0] versus 21.6 [19.2–24.2] kg/m^2^) and higher mean serum albumin levels (3.5 [SD, 0.3] versus 3.2 [0.3] g/dl) compared with the conventional hemodialysis group. In both groups, approximately 50% of the patients had diabetes mellitus. Although the mean predialysis BP was higher in the extended-hours hemodialysis group, the proportion of antihypertensive medication use was lower than that in the conventional hemodialysis group. The extended-hours hemodialysis group also showed lower use of erythropoiesis-stimulating agents, phosphate binders, and active vitamin D than the conventional hemodialysis group. The mean single-pool Kt/V_urea_ values were 1.79 for the extended-hours hemodialysis group and 1.63 for the conventional hemodialysis group. There was no significant difference in the mean nPCR between the two groups (0.89 g/kg per day for extended-hours hemodialysis versus 0.90 g/kg per day for conventional hemodialysis).

**Table 1:** Baseline characteristics of patients on extended-hours and those on conventional hemodialysis

Characteristics	Extended-Hours Hemodialysis (*n*=188)	Conventional Hemodialysis (*n*=286)
Age, yr	66±12	69±12
Male, No. (%)	131 (70)	193 (68)
BMI, kg/m^2^	22.8 (21.0–26.0)	21.6 (19.2–24.2)
Time since starting dialysis, yr	9.1 (3.4–15.8)	6.0 (2.5–13.3)
Extended-hours duration on dialysis, yr	8.0 (2.8–13.6)	0
Dialysis hours per week, h	21.9±3.2	12.1±1.1
Blood flow rate, ml/min	153±23	243±32
**Dialysis technique, No. (%)**		
Hemodialysis	184 (98)	67 (23)
HDF	4 (2)	219 (77)
High-flux dialyzer	188 (100)	280 (98)
Low-flux dialyzer	0 (0)	6 (2)
spKt/V^a^	1.79±0.41	1.63±0.29
nPCR^b^, g/kg per day	0.89±0.19	0.90±0.17
**Comorbidities, No. (%)**		
Diabetes mellitus	91 (48)	143 (50)
Hypertension	87 (46)	240 (84)
Coronary artery disease	41 (22)	87 (30)
Peripheral artery disease	27 (14)	80 (28)
Cerebrovascular disease	48 (26)	52 (18)
Cancer	3 (1.6)	8 (2.8)
**Laboratory data**		
Serum albumin, g/dl	3.5±0.3	3.2±0.3
Serum creatinine, mg/dl	10.0±2.5	10.2±2.5
Serum calcium, mg/dl	8.5±0.6	8.4±0.6
Serum phosphate, mg/dl	5.4±1.3	5.4±1.1
Serum intact PTH, pg/ml	188 (123–269)	193 (120–270)
Serum C-reactive protein, mg/dl	0.09 (0.04–0.29)	0.14 (0.05–0.38)
Serum *β*2-microglobulin, mg/l	25.3±4.6	28.1±6.5
**Medications, No. (%)**		
Antihypertensives	88 (47)	239 (84)
Erythropoiesis-stimulating agents	117 (62)	240 (84)
Phosphate binders	104 (55)	249 (87)
Active vitamin D	105 (56)	232 (81)
Calcimimetics	77 (41)	109 (38)
Predialysis systolic BP, mm Hg	156±29	142±25
Predialysis diastolic BP, mm Hg	84±17	75±14
Postdialysis systolic BP, mm Hg	131±25	138±25
Postdialysis diastolic BP, mm Hg	74±16	78±14

The table presents participants’ characteristics as mean±SD or median (interquartile range) for continuous variables and No. (%) for categorical variables. BMI, body mass index; HDF, hemodiafiltration; nPCR, normalized protein catabolic rate; PTH, parathyroid hormone; spKt/V, single-pool Kt/V_urea_.

Pre- and postdialysis BP values were measured on the day of blood sample collection.

a Shows values excluding some missing values (extended-hours hemodialysis: *n*=184, conventional hemodialysis: *n*=286).

b Shows values excluding some missing values (extended-hours hemodialysis: *n*=185, conventional hemodialysis: *n*=281).

**Figure 1:**
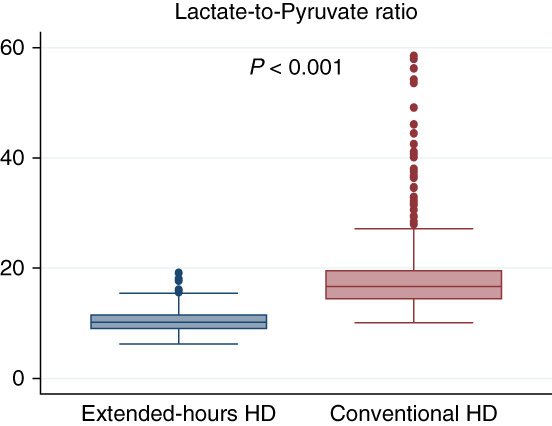
**Box plot showing the comparison of the lactate-to-pyruvate ratio between the extended-hours and conventional hemodialysis groups.** *P* values were derived from the Wilcoxon rank-sum test. HD, hemodialysis.

In the plasma samples collected in our study, 272 cationic and 236 anionic metabolites were quantified. The Wilcoxon rank-sum tests showed statistically significant differences in 39 of 117 analyzed metabolites between the two groups; 18 were higher in the extended-hours hemodialysis group, and 21 were higher in the conventional hemodialysis group (Table 2). Regarding known uremic toxins, uridine and hypoxanthine levels were significantly higher in the conventional hemodialysis group than in the extended-hours hemodialysis group (Table 3). The lactate-to-pyruvate ratio was significantly lower in the extended-hours hemodialysis group compared with the conventional hemodialysis group (10.2 [8.9–11.6] versus 16.7 [14.3–19.6]; Figure 1). The levels of plasma essential amino acids, including BCAAs (valine, leucine, isoleucine), lysine, methionine, threonine, and histidine, were significantly higher in the extended-hours hemodialysis group than in the conventional hemodialysis group (Table 3). The levels of plasma nonessential amino acids, including arginine and asparagine, were higher in the extended-hours hemodialysis group than in the conventional hemodialysis group, whereas aspartic acid and glutamic acid levels were lower in the extended-hours hemodialysis group than in the conventional hemodialysis group.

**Table 2:** Comparison of metabolites with statistical superiority across different dialysis methods

Metabolites, *μ*M	Extended-Hours Hemodialysis	Conventional Hemodialysis	*P* Value
Uridine	48.3 (43.1–52.8)	60.0 (54.7–65.3)	5.6×10^−42^
Hypoxanthine	3.31 (2.61–3.90)	5.15 (4.31–6.27)	1.8×10^−32^
Pelargonate	2.12 (1.84–2.34)	3.90 (2.26–4.53)	2.6×10^−24^
Ethanolamine phosphate	2.69 (2.21–3.20)	4.34 (3.18–5.61)	3.3×10^−24^
Decanoate	0.96 (0.83–1.12)	2.68 (2.37–3.15)	7.4×10^−24^
Aspartic acid	4.59 (3.73–5.40)	5.98 (5.08–7.26)	3.2×10^−23^
5-oxoproline	43.2 (38.5–47.7)	52.9 (46.1–60.4)	8.8×10^−23^
Lactate	852 (719–1056)	1148 (951–1373)	5.3×10^−21^
Terephthalate	0.74 (0.58–0.88)	2.13 (0.71–2.91)	1.7×10^−19^
Glycerophosphate	2.68 (2.29–3.33)	1.89 (1.53–2.43)	3.8×10^−19^
3-Phosphoglycerate	0.88 (0.71–1.23)	2.00 (1.45–2.67)	9.4×10^−19^
Adenosine diphosphate	0.39 (0.28–0.46)	1.60 (1.03–2.25)	8.5×10^−15^
Histidine	71.9 (63.9–79.6)	61.9 (54.4–71.7)	1.6×10^−14^
Ornithine	75.9 (61.8–92.1)	93.3 (76.9–111.6)	2.2×10^−14^
Phthalate	0.28 (0.23–0.40)	0.48 (0.39–0.58)	3.9×10^−12^
Asparagine	59.1 (52.1–68.6)	50.9 (44.2–59.4)	4.8×10^−12^
4-acetylbutyrate	1.86 (1.62–2.17)	3.25 (1.71–3.80)	5.7×10^−12^
Pyruvate	83.7 (67.1–103.9)	65.9 (52.7–84.3)	1.0×10^−11^
Allantoate	6.89 (5.00–9.25)	4.88 (3.64–6.79)	1.3×10^−10^
Methionine sulfoxide	3.18 (2.64–3.75)	2.68 (2.22–3.19)	4.0×10^−10^
Valine	190 (164–220)	167 (145–198)	6.3×10^−9^
Methionine	19.8 (16.6–23.5)	16.9 (14.3–20.7)	1.6×10^−8^
Glutamic acid	55.2 (41.4–73.9)	66.8 (55.3–84.6)	2.9×10^−8^
Cis-aconitate	12.41 (10.56–15.28)	11.18 (8.54–13.44)	2.1×10^−7^
Threonate	103.5 (86.0–121.6)	117.8 (97.2–138.4)	3.1×10^−7^
Isoleucine	68.8 (56.9–82.0)	60.4 (46.2–71.5)	4.4×10^−7^
2-aminobutanoate	9.89 (7.95–12.27)	8.19 (6.53–10.84)	4.5×10^−7^
Threonine	127.0 (107.2–156.7)	110.9 (89.9–137.3)	6.2×10^−7^
Leucine	100.9 (81.8–121.8)	87.3 (69.2–105.8)	6.7×10^−7^
Lysine	169 (145–194)	152 (127–175)	1.6×10^−6^
*α*-aminoadipate	1.55 (1.19–2.06)	1.29 (1.00–1.67)	4.8×10^−6^
Tryptophol	4.09 (3.66–4.69)	5.16 (4.13–6.26)	7.7×10^−6^
Succinate	9.49 (7.71–12.22)	11.11 (9.17–13.8)	9.1×10^−6^
2-hydroxyisobutyrate	3.81 (3.13–4.69)	4.41 (3.56–5.27)	9.5×10^−6^
Choline	36.0 (31.1–41.7)	32.4 (24.2–40.0)	1.4×10^−5^
Alanine	426 (353–501)	382 (322–446)	2.0×10^−5^
Cysteine S-sulfate	1.28 (0.99–1.49)	1.43 (1.13–1.73)	1.4×10^−4^
Taurine	73.2 (53.0–109.8)	89.5 (67.4–118.9)	2.3×10^−4^
Arginine	96.0 (81.4–117.2)	87.4 (73.0–105.3)	4.2×10^−4^

Values are presented as medians (interquartile ranges) for continuous variables. Listed 39 metabolites were significantly different between the extended-hours and conventional hemodialysis groups. *P* values were derived from the Wilcoxon rank-sum test and were significant at the Bonferroni-adjusted level of 4.3×10^−4^.

**Table 3:** Comparison of uremic solutes and branched-chain amino acids across different dialysis methods

Metabolite, *μ*M	Extended-Hours Hemodialysis	Conventional Hemodialysis	*P* Value	Difference (Extended-Hours Hemodialysis versus Conventional Hemodialysis)	Adjusted *P*
**Uremic solutes**					
Indoxyl sulfate	153.5 (107.5–198.1)	132.6 (91.8–185.1)	0.03	11.8 (−2.7 to 26.4)	0.11
Indole-3-acetate	7.96 (6.31–10.66)	7.80 (6.29–9.60)	0.34	0.99 (−0.59 to 2.57)	0.22
TMAO	132.7 (86.4–198.9)	109.1 (73.9–154.1)	0.0006	32.5 (12.8 to 52.2)	0.001^a^
ADMA	1.07 (0.93–1.24)	1.10 (0.97–1.26)	0.12	−0.02 (−0.08 to 0.03)	0.44
Hippurate	144.4 (75.1–256.6)	138.8 (70.4–208.7)	0.13	20.0 (−7.7 to 47.8)	0.16
Uridine	48.3 (43.1–52.8)	60.0 (54.7–65.3)	<0.0001^b^	−11.0 (−12.7 to −9.3)	<0.0001^a^
Hypoxanthine	3.31 (2.61–3.90)	5.15 (4.31–6.27)	<0.0001^b^	−2.38 (−2.85 to −1.91)	<0.0001^a^
*N*,*N*-dimethylglycine	10.57 (8.69–12.46)	11.27 (8.79–13.80)	0.08	−0.99 (−1.87 to −0.11)	0.03
Guanidinosuccinate	8.37 (5.69–12.29)	9.02 (6.13–12.88)	0.21	−0.02 (−1.05 to 1.02)	0.98
Kynurenine	3.06 (2.52–3.80)	2.79 (2.30–3.47)	0.005	0.26 (0.04 to 0.48)	0.02
SDMA	4.16 (2.90–7.62)	3.83 (2.97–5.38)	0.17	0.73 (−0.06 to 1.53)	0.07
Urea	10,813 (9139–12441)	11,019 (9286–11265)	0.80	276 (−219 to 771)	0.27
Creatine	26.5 (17.3–54.1)	26.1 (17.1–44.1)	0.46	9.8 (−5.7 to 25.2)	0.21
Creatinine	756 (601–856)	751 (627–916)	0.30	−9 (−41 to 24)	0.61
**BCAAs**					
Valine	190 (164–220)	167 (145–198)	<0.0001^b^	17.7 (8.1 to 27.4)	0.0003^c^
Leucine	100.9 (81.8–121.8)	87.3 (69.2–105.8)	<0.0001^b^	9.6 (3.3 to 15.9)	0.0027^c^
Isoleucine	68.8 (56.9–82.0)	60.4 (46.2–71.5)	<0.0001^b^	6.9 (3.0 to 10.7)	0.0006^c^

Between-group comparisons were performed using the Wilcoxson rank-sum test. Values are presented as medians (interquartile ranges) or differences (95% confidence intervals). Differences in metabolites between extended-hours and conventional hemodialysis were calculated by propensity score–adjusted multiple regression analysis. Propensity scores were estimated using the following covariates: age, sex, time since starting dialysis, body mass index, diabetes mellitus, hypertension, coronary artery disease, peripheral artery disease, and cerebrovascular disease. Multiple regression analysis was adjusted for propensity scores, propensity score model variables, plus medication data (*i.e*., use of phosphate binders, active vitamin D, and calcimimetics). ADMA, asymmetric dimethylarginine; BCAA, branched-chain amino acid; SDMA, symmetric dimethylarginine; TMAO, trimethylamine *N*-oxide.

a Adjusted *P* values were considered significant at the Bonferroni-adjusted level of 3.6×10^−3^.

b *P* values were considered significant at the Bonferroni-adjusted level of 4.3×10^−4^.

c Adjusted *P* values were considered significant at the Bonferroni-adjusted level of 0.017.

**Figure 2:**
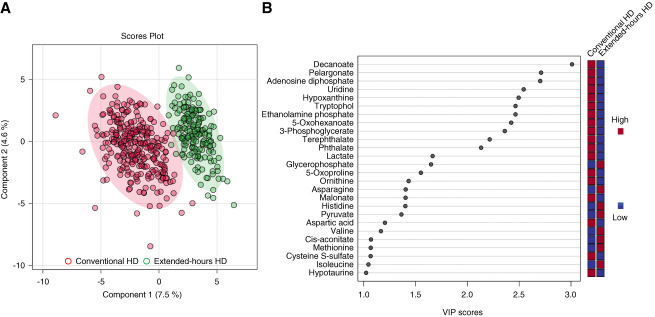
**Comparison of plasma metabolic profiles across different dialysis methods by PLS-DA.** (A) PLS-DA score plot. Participants were grouped according to extended-hours (green) and conventional (red) hemodialysis. The score plot displays the separation between the two groups. Colored ellipses illustrate the 95% CIs. Colored dots represent individual samples. (B) The VIP scores estimated the importance of metabolites in the projection used in the PLS model. In total, 26 metabolites had VIP scores ≥1.0. The colored boxes on the right indicate the direction of change in the corresponding metabolite in the dialysis method. CI, confidence interval; PLS-DA, partial least squares discriminant analysis; VIP, variable importance for projection.

**Figure 3:**
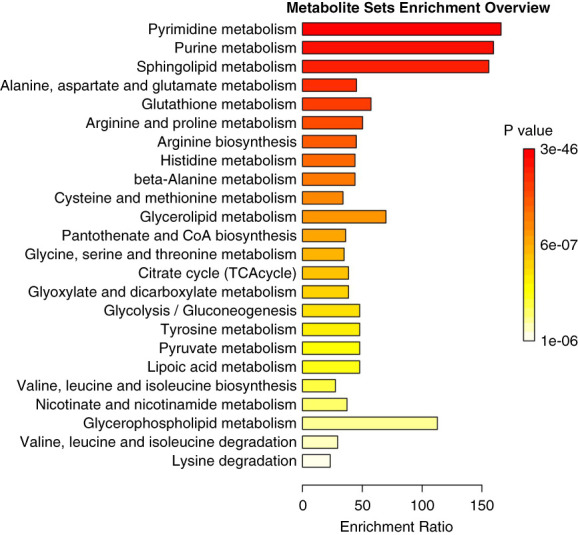
**Summary plot for QEA on the basis of the KEGG database.** QEA was performed on 24 metabolites that were statistically significant by both the Wilcoxon rank-sum test and PLS-DA. KEGG pathways represent all statistically significant pathways shown in the bar chart, and the enrichment ratio was computed as observed hits/expected hits. CoA, coenzyme A KEGG, Kyoto Encyclopedia of Genes and Genomes; QEA, quantitative enrichment analysis; TCA, tricarboxylic acid.

Although PCA did not adequately discriminate plasma metabolic profiles between the extended-hours and conventional hemodialysis groups with low principal component contribution rates (Supplemental Figure 1), PLS-DA appropriately discriminated the plasma metabolic profiles between the two groups (Figure 2A). PLS-DA identified 26 metabolites with VIP scores ≥1.0 (Figure 2B), 24 of which (excluding malonate and hypotaurine) were consistent with the results of the Wilcoxon rank-sum test. QEA performed on 24 metabolites demonstrated enriched pathways, particularly in pyrimidine, purine, and sphingolipid metabolisms (Figure 3). Significant differences were also observed in glycolysis/gluconeogenesis, pyruvate metabolism, and tricarboxylic acid cycle, which are related to lactate and pyruvate metabolism, between the two groups.

**Figure 4:**
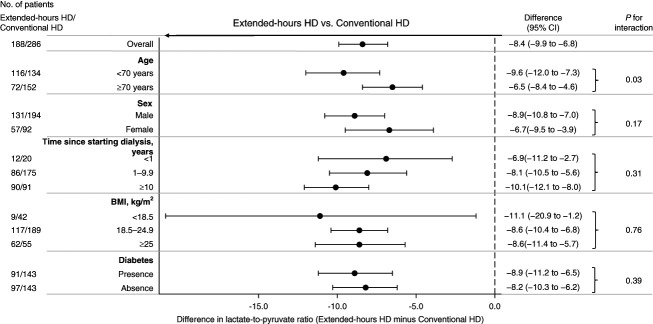
**Forest plot showing adjusted differences and 95% CIs for lactate-to-pyruvate ratio in patients on extended-hours hemodialysis compared with conventional hemodialysis stratified by subgroups.** The estimated difference was adjusted for multiple regression analysis using the following covariates: age, sex, time since starting dialysis, BMI, diabetes mellitus, hypertension, coronary artery disease, peripheral artery disease, cerebrovascular disease, and medications (use of phosphate binders, active vitamin D, and calcimimetics). BMI, body mass index.

In the PS-adjusted multiple regression analysis, uridine and hypoxanthine levels were significantly higher in the conventional hemodialysis group than in the extended-hours hemodialysis group, whereas trimethylamine-*N*-oxide (TMAO) level was higher in the extended-hours hemodialysis group than in the conventional hemodialysis group (Table 3). All BCAA levels remained significantly higher in the extended-hours hemodialysis group compared with the conventional hemodialysis group. There were no statistically significant differences between the two groups in the levels of gut-derived uremic retention solutes, such as indoxyl sulfate and hippurate. The lactate-to-pyruvate ratio remained statistically lower in the extended-hours hemodialysis group with a significant difference of −8.6 (95% confidence interval, −9.8 to −7.4) compared with the conventional hemodialysis group (Supplemental Table 3). The subgroup analysis of the lactate-to-pyruvate ratio revealed a consistent trend of lower values in the extended-hours hemodialysis group than in the conventional hemodialysis group across all subgroups (Figure 4). The difference in the lactate-to-pyruvate ratio was particularly larger in patients younger than 70 years with statistically significant interaction (*P*_interaction_ = 0.03). The difference in the lactate-to-pyruvate ratio tended to be larger with a longer time since starting dialysis without statistical significance. For the fit of IPTW, the C-statistic of the logistic regression analysis was 0.78, and the absolute standardized differences of the covariates were all <0.1 (Supplemental Table 4).

**Figure 5:**
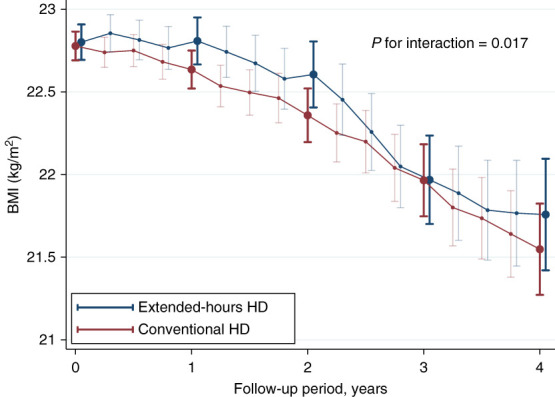
**Estimated trajectories of BMI stratified by dialysis methods.** The estimated BMI was calculated using a linear mixed-effects model adjusted for baseline age, sex, BMI, diabetes mellitus, and time since starting dialysis. This model included random intercepts and slopes for each patient and accounted for within-patient correlation using an unstructured variance–covariance matrix.

The Kaplan–Meier survival curves showed a trend toward better survival outcomes in the extended-hours hemodialysis group compared with the conventional hemodialysis group; however, the difference was not statistically significant (log-rank test, *P* = 0.072; Supplemental Figure 2). The estimated BMI trajectory showed a gradual decline over time; however, BMI tended to be significantly better maintained in the extended-hours hemodialysis group than in the conventional hemodialysis group (*P*_interaction_ = 0.017; Figure 5).

## Discussion

In this metabolomic analysis using CE-TOFMS with the largest multicenter cohort of patients on in-center daytime extended-hours hemodialysis in Japan, we detected 117 plasma metabolites with measurement values of ≥70% in either extended-hours or conventional hemodialysis. Among those, 39 metabolites showed statistically significant differences between the two groups. There was no statistically significant difference in the established protein-bound uremic toxin of indoxyl sulfate; however, some known uremic retention solutes showed higher levels in the conventional hemodialysis group than in the extended-hours hemodialysis group. The lactate-to-pyruvate ratio, a possible surrogate marker of mitochondrial dysfunction, was significantly lower in the extended-hours hemodialysis group than the conventional hemodialysis group, suggesting better metabolic conditions in the extended-hours hemodialysis group. In addition, patients on extended-hours hemodialysis showed higher levels of seven essential plasma amino acids, including all BCAAs, than those on conventional hemodialysis.

A previous study has shown important findings regarding changes in concentrations of uremic retention solutes in patients on extended-hours hemodialysis performed overnight (*i.e*., nocturnal hemodialysis) in hospital; however, enrolled patients in previous studies were in their 50s and had a BMI of >25 kg/m^2^, which may be associated with low susceptibility to the protein–energy malnutrition.^17^ This study included in-center daytime outpatients who were similar to Japanese patients on maintenance hemodialysis, with a mean age of 68.8 years.^35^ This large multicenter Japanese cohort study can provide new insight into the metabolic and nutritional disorders in patients on maintenance hemodialysis.

Uremic toxins are defined as compounds of uremic retention solutes that are abnormally elevated in blood and have negative biological or clinical effects in patients with CKD.^7^ Among the known 14 uremic toxins, which include highly toxic water-soluble small molecule toxins (asymmetric dimethylarginine, symmetric dimethylarginine, TMAO, and urea) and protein-bound toxins (indoxyl sulfate, indole acetic acid, kynurenines, and hippurate),^36^ only uridine and hypoxanthine levels were significantly higher in conventional hemodialysis on the basis of univariate analysis and PLS-DA. Toxicity studies on uridine and hypoxanthine have reported that they are associated with decreased eGFR^37^ and inhibition of calcitriol (1,25-[OH]_2_D_3_) synthesis,^36,38^ respectively. Uridine and hypoxanthine, both water-soluble small molecule toxins, are metabolites of the pyrimidine and purine metabolic pathways, respectively. Given the high enrichment ratios in QEA, these pathways may be particularly susceptible to the influences of extended-hours hemodialysis or dietary factors. In multiple regression analysis, TMAO levels were significantly higher in the extended-hours hemodialysis group than in the conventional hemodialysis group. TMAO is a small amine plasma compound produced by choline metabolism in the gut microbiota and has been implicated in increased cardiovascular risk.^39^ Plasma TMAO concentration can be directly influenced by dietary habits, gut dysbiosis related to uremic milieu, and dietary restrictions. One possible explanation for our finding is that the liberalized dietary habits, allowed by the longer dialysis treatment time, were related to TMAO production. This hypothesis is based on the knowledge that TMAO is a small solute that is easily removed by dialysis and on our finding that single-pool Kt/V urea, which shows similar solute clearance of TMAO, was relatively similar in extended-hours hemodialysis with a low blood flow rate compared with conventional hemodialysis.^40,41^ Protein-bound uremic toxins are difficult to remove regardless of the adequate parameter Kt/V_urea_ by increasing the blood flow rate.^42^ Two-compartment kinetic modeling has suggested that frequent and/or extended-hours hemodialysis may enhance the removal of various protein-bound uremic retention solutes.^43^ However, previous studies have shown only a slight decrease in uremic toxins with 6-times weekly in-center hemodialysis and no significant change with extended-hours nocturnal hemodialysis.^17,44^ This study did not show significant differences in the concentration of protein-bound uremic toxins. Even with an increased amount of uremic solute removal, an increased dietary intake of protein-containing meals may promote the production of uremic toxins.

Mitochondria play a central role in maintaining skeletal muscle by supplying energy through oxidative phosphorylation.^45^ Patients on dialysis often experience mitochondrial dysfunction because of factors such as uremia, malnutrition, and oxidative stress, contributing to protein–energy wasting pathogenesis.^46,47^ The lactate-to-pyruvate ratio is clinically used as one of the biomarkers of mitochondrial dysfunction, including chronic metabolic abnormalities.^20^ In this study, the lactate-to-pyruvate ratio was increased in conventional hemodialysis, but remained within the normal range in extended-hours hemodialysis. In patients on conventional hemodialysis, there is a potential increase in the lactate-to-pyruvate ratio, possibly owing to compromised energy production in skeletal muscles through impaired aerobic metabolism, which is compensated for by an increase in anaerobic glycolysis.^48^ Conversely, the possibility of an improved lactate-to-pyruvate ratio in extended-hours hemodialysis may result from improved aerobic metabolism in mitochondria-rich tissues, such as skeletal muscle, because there were significant differences between the two groups in the tricarboxylic acid cycle and glycolytic pathways in the QEA. Further studies are warranted to evaluate the status of skeletal muscles during extended-hours hemodialysis for validation. Subgroup analyses showed consistent increases in the lactate-to-pyruvate ratio in the conventional hemodialysis group, suggesting that extended-hours hemodialysis may be a therapeutic approach with the potential to improve mitochondrial function.

BCAAs, which are essential amino acids dependent on dietary intake, are the building blocks of protein synthesis in skeletal muscles.^49^ A previous study on 8-hour nocturnal hemodialysis found significantly elevated plasma BCAA concentrations despite the potential for increased amino acid loss during longer dialysis treatment.^17^ Our study also found that the plasma concentrations of seven of nine essential amino acids, including all BCAAs, were significantly higher in extended-hours hemodialysis than in conventional hemodialysis. These results suggest that the effects of protein intake and synthesis may exceed those of amino acid loss in extended-hours hemodialysis, leading to an improved plasma amino acid profile.^50^ There are several possible reasons for the lack of differences in the nPCR, a measure of protein intake, although most plasma essential amino acid concentrations were higher in extended-hours hemodialysis than in conventional hemodialysis. First, extended-hours hemodialysis may decrease nPCR by increasing postdialysis blood urea nitrogen levels, which could be influenced by both dietary consumption during dialysis^51^ and a lower blood flow rate. In addition, the calculation of nPCR considers endogenous protein catabolism,^52^ but the state of catabolism may vary owing to differences in the dialysis method. The 4-year BMI trajectories and survival curves suggest that extended-hours hemodialysis may suppress weight loss over the long term compared with conventional hemodialysis, indicating a tendency for better survival outcomes. These findings suggest that the favorable metabolic and nutritional profiles observed in this study have clinical relevance.

This study has some limitations. First, this was a cross-sectional study and did not establish causation. However, considering the context of this study, reverse causation is unlikely to occur. Second, there is a potential for selection bias concerning the choice of dialysis modality and confounding bias because of differences in baseline nutritional status influenced by factors such as comorbidities. Although multivariable adjustment using the IPTW method was used to mitigate these biases, it is possible that residual confounding may not have been fully adjusted for. Third, the nature of metabolomic analysis imposes constraints on the comprehensive measurement of all metabolites. For instance, it is difficult to assess metabolites, such as middle-molecular-weight uremic toxins and small molecule metabolites, with missing values. The metabolites not measured in this study warrant further evaluation. Finally, the presence of a favorable metabolic profile does not necessarily indicate a reduction in skeletal muscle loss or cachexia; therefore, future clinical trials are needed to evaluate the effect of extended-hours hemodialysis on outcomes related to protein–energy wasting.

In conclusion, this study revealed differences in the plasma metabolome profiles between extended-hours and conventional hemodialysis. Extended-hours hemodialysis was associated with a lower lactate-to-pyruvate ratio, suggesting improved metabolic conditions and increased essential amino acid levels, including BCAAs. Although the concentrations of most uremic retention solutes were not significantly different, our findings suggest the potential benefits of extended-hours hemodialysis for improving metabolic and nutritional profiles among patients treated with maintenance hemodialysis.

## Supplementary Material

SUPPLEMENTARY MATERIAL

## Data Availability

Partial restrictions to the data and/or materials apply. The data supporting the findings of this study are not publicly available because they contain information that could compromise the privacy of research participants. Deidentified data can be provided upon reasonable request to the corresponding author.

## Supplemental Material

Supplemental Figure 1. Comparison of metabolite profiles on different dialysis methods by PCA.

Supplemental Figure 2. Kaplan–Meier analysis of all-cause mortality of patients on extended-hours and those on conventional hemodialysis.

Supplemental Table 1. Nutrition content of meals provided during extended-hours hemodialysis.

Supplemental Table 2. List of 117 metabolites included in the statistical analyses.

Supplemental Table 3. Comparison of lactate-to-pyruvate ratio across different dialysis methods.

Supplemental Table 4. Fitting of inverse probability of treatment weighting propensity score.

Supplemental Method 1. Brief description of the sample preparation and measurement process.
