# Supplementary material for: Plasma Metabolite Profiles Between In-Center Daytime Extended-Hours and Conventional Hemodialysis
Source: Kidney360. 2024 Dec 9;6(3):420–31. doi: 10.34067/KID.0000000675 (PMC11970860; doi:10.34067/KID.0000000675)
Supplement: SUPPLEMENTARY MATERIAL [file kidney360-6-420-s001.pdf]

ASN American Society of Nephrology | JASN Journal of the American Society of Nephrology | KIDNEY360 Accessing Our World From Every Angle | CJASN Clinical Journal of the American Society of Nephrology

ASN Journal Disclosure Form

As per ASN journal policy, I have disclosed any financial relationships or commitments I have held in the past 36 months as included below. I have listed my Current Employer below to indicate there is a relationship requiring disclosure. If no relationship exists, my Current Employer is not listed.

S. Akiyama reports the following:
Employer: Nagoya University Graduate School of Medicine; and Research Funding: Alexion; Asahi Kasei Pharma; Astellas Pharma Inc.; Baxter; Bristol-Myers Squibb; Chugai Pharmaceutical Co., Ltd; Daiichi Sankyo Co., Ltd; Kyowa Hakko Kirin Co., Ltd; Mercj Sharp and Dohme; Mitsubishi Tanabe Pharma Co.; Mochida Pharmaceutical Co., Ltd; Novartis Pharma; Otsuka Pharmaceutical Co., Ltd; Pfizer Japan Inc.; Sanwa Kagaku Kenkyusho Co., Ltd; Sumitomo Dainippon Pharma Co., Ltd; Takeda Pharmaceutical Co., Ltd; Teijin Pharma Ltd; Torii Pharmaceutical Co., Ltd.

I understand that the information above will be published within the journal article, if accepted, and that failure to comply and/or to accurately and completely report the potential financial conflicts of interest could lead to the following: 1) Prior to publication, article rejection, or 2) Post-publication, sanctions ranging from, but not limited to, issuing a correction, reporting the inaccurate information to the authors’ institution, banning authors from submitting work to ASN journals for varying lengths of time, and/or retraction of the published work.

Name: Shin'ichi Akiyama
Manuscript ID: K360-2024-000725R1
Manuscript Title: Plasma metabolite profiles between in-center daytime extended-hours and conventional hemodialysis
Date of Completion: November 5, 2024
Disclosure Updated Date: November 5, 2024

ASN
American Society of Nephrology
JASN
Journal of the American Society of Nephrology
KIDNEY360
Accessing Our World From Every Angle
CJASN
Clinical Journal of the American Society of Nephrology

ASN Journal Disclosure Form

As per ASN journal policy, I have disclosed any financial relationships or commitments I have held in the past 36 months as included below. I have listed my Current Employer below to indicate there is a relationship requiring disclosure. If no relationship exists, my Current Employer is not listed.

A. Hirayama reports the following:
Employer: Keio University; and Ownership Interest: INCEMS Technologies, Co. Ltd.

I understand that the information above will be published within the journal article, if accepted, and that failure to comply and/or to accurately and completely report the potential financial conflicts of interest could lead to the following: 1) Prior to publication, article rejection, or 2) Post-publication, sanctions ranging from, but not limited to, issuing a correction, reporting the inaccurate information to the authors' institution, banning authors from submitting work to ASN journals for varying lengths of time, and/or retraction of the published work.

Name: Akiyoshi Hirayama
Manuscript ID: K360-2024-000725R1
Manuscript Title: Plasma metabolite profiles between in-center daytime extended-hours and conventional hemodialysis.
Date of Completion: November 5, 2024
Disclosure Updated Date: November 5, 2024

ASN American Society of Nephrology JASN Journal of the American Society of Nephrology KIDNEY360 Accessing Our World From Every Angle CJASN Clinical Journal of the American Society of Nephrology

ASN Journal Disclosure Form

As per ASN journal policy, I have disclosed any financial relationships or commitments I have held in the past 36 months as included below. I have listed my Current Employer below to indicate there is a relationship requiring disclosure. If no relationship exists, my Current Employer is not listed.

M. Hishida reports the following:
Employer: Kaikoukai Josai Hospital; and Other Interests or Relationships: Japanese Society of Nephrology, The Japanese Society for Dilysis therapy.

I understand that the information above will be published within the journal article, if accepted, and that failure to comply and/or to accurately and completely report the potential financial conflicts of interest could lead to the following: 1) Prior to publication, article rejection, or 2) Post-publication, sanctions ranging from, but not limited to, issuing a correction, reporting the inaccurate information to the authors' institution, banning authors from submitting work to ASN journals for varying lengths of time, and/or retraction of the published work.

Name: Manabu Hishida
Manuscript ID: K360-2024-000725R1
Manuscript Title: Plasma metabolite profiles between in-center daytime extended-hours and conventional hemodialysis
Date of Completion: November 7, 2024
Disclosure Updated Date: November 7, 2024

ASN American Society of Nephrology | JASN Journal of the American Society of Nephrology | KIDNEY360 Accessing Our World From Every Angle | CJASN Clinical Journal of the American Society of Nephrology

ASN Journal Disclosure Form

As per ASN journal policy, I have disclosed any financial relationships or commitments I have held in the past 36 months as included below. I have listed my Current Employer below to indicate there is a relationship requiring disclosure. If no relationship exists, my Current Employer is not listed.

T. Imaizumi reports the following:
Consultancy: GlaxoSmithKline; Research Funding: Kyowa Kirin Co., Ltd.; and Honoraria: Kyowa Kirin Co., Ltd.

I understand that the information above will be published within the journal article, if accepted, and that failure to comply and/or to accurately and completely report the potential financial conflicts of interest could lead to the following: 1) Prior to publication, article rejection, or 2) Post-publication, sanctions ranging from, but not limited to, issuing a correction, reporting the inaccurate information to the authors' institution, banning authors from submitting work to ASN journals for varying lengths of time, and/or retraction of the published work.

Name: Takahiro Imaizumi
Manuscript ID: K360-2024-000725R1
Manuscript Title: Plasma metabolite profiles between in-center daytime extended-hours and conventional hemodialysis
Date of Completion: November 5, 2024
Disclosure Updated Date: November 5, 2024

ASN Journal Disclosure Form

As per ASN journal policy, I have disclosed any financial relationships or commitments I have held in the past 36 months as included below. I have listed my Current Employer below to indicate there is a relationship requiring disclosure. If no relationship exists, my Current Employer is not listed.

F. Kaneda reports the following:
Employer: kamome clinic

I understand that the information above will be published within the journal article, if accepted, and that failure to comply and/or to accurately and completely report the potential financial conflicts of interest could lead to the following: 1) Prior to publication, article rejection, or 2) Post-publication, sanctions ranging from, but not limited to, issuing a correction, reporting the inaccurate information to the authors' institution, banning authors from submitting work to ASN journals for varying lengths of time, and/or retraction of the published work.

Name: Fumika Kaneda
Manuscript ID: K360-2024-000725R1
Manuscript Title: Plasma metabolite profiles between in-center daytime extended-hours and conventional hemodialysis
Date of Completion: November 6, 2024
Disclosure Updated Date: November 6, 2024

ASN American Society of Nephrology · JASN Journal of the American Society of Nephrology · KIDNEY360 Accessing Our World From Every Angle · CJASN Clinical Journal of the American Society of Nephrology

ASN Journal Disclosure Form

As per ASN journal policy, I have disclosed any financial relationships or commitments I have held in the past 36 months as included below. I have listed my Current Employer below to indicate there is a relationship requiring disclosure. If no relationship exists, my Current Employer is not listed.

H. Kasuga reports the following:
Employer: Nagoya Kyoritsu Hospital

I understand that the information above will be published within the journal article, if accepted, and that failure to comply and/or to accurately and completely report the potential financial conflicts of interest could lead to the following: 1) Prior to publication, article rejection, or 2) Post-publication, sanctions ranging from, but not limited to, issuing a correction, reporting the inaccurate information to the authors' institution, banning authors from submitting work to ASN journals for varying lengths of time, and/or retraction of the published work.

Name: Hirotake Kasuga
Manuscript ID: K360-2024-000725R1
Manuscript Title: Plasma metaolite profiles between in-center daytime extended-hours and convetional hemodialysis
Date of Completion: November 6, 2024
Disclosure Updated Date: November 6, 2024

ASN American Society of Nephrology
JASN Journal of the American Society of Nephrology
KIDNEY360 Accessing Our World From Every Angle
CJASN Clinical Journal of the American Society of Nephrology

ASN Journal Disclosure Form

As per ASN journal policy, I have disclosed any financial relationships or commitments I have held in the past 36 months as included below. I have listed my Current Employer below to indicate there is a relationship requiring disclosure. If no relationship exists, my Current Employer is not listed.

S. Kurasawa reports the following:
Honoraria: Kyowa Kirin Co., Ltd and Mochida Pharmaceutical Co., Ltd.

I understand that the information above will be published within the journal article, if accepted, and that failure to comply and/or to accurately and completely report the potential financial conflicts of interest could lead to the following: 1) Prior to publication, article rejection, or 2) Post-publication, sanctions ranging from, but not limited to, issuing a correction, reporting the inaccurate information to the authors' institution, banning authors from submitting work to ASN journals for varying lengths of time, and/or retraction of the published work.

Name: Shimon Kurasawa
Manuscript ID: K360-2024-000725R1
Manuscript Title: Plasma metabolite profiles between in-center daytime extended-hours and conventional hemodialysis
Date of Completion: November 6, 2024
Disclosure Updated Date: November 6, 2024

ASN Journal Disclosure Form

As per ASN journal policy, I have disclosed any financial relationships or commitments I have held in the past 36 months as included below. I have listed my Current Employer below to indicate there is a relationship requiring disclosure. If no relationship exists, my Current Employer is not listed.

S. Maruyama reports the following:
Ownership Interest: Zome Co.; Research Funding: Chugai Pharmaceutical Co. Ono Pharmaceutical Co., Ltd. Rohto Pharmaceutical Co.,Ltd. Mitsubishi Tanabe Pharma Corp. Mitsubishi Foundation, Sanofi K.K., Baxter Limited., Pfizer Japan Inc. Kyowa Kirin Co ., Ltd. Otsuka Pharmaceutical Co. Sumitomo, Dainippon Pharma Co., Ltd., Chugai Pharmaceutical Co., Torii Pharmaceutical Co.,Ltd., Teijin Pharma Limited. ;; and Honoraria: Chugai Pharmaceutical Co., Ltd, Alexion Pharmaceuticals, Inc, Mitsubishi Tanabe Pharma Corp., Bayer Pharma Japan, Astellas Pharma Inc., AstraZeneca K.K., Ono Pharmaceutical Co ., Ltd ;.

I understand that the information above will be published within the journal article, if accepted, and that failure to comply and/or to accurately and completely report the potential financial conflicts of interest could lead to the following: 1) Prior to publication, article rejection, or 2) Post-publication, sanctions ranging from, but not limited to, issuing a correction, reporting the inaccurate information to the authors' institution, banning authors from submitting work to ASN journals for varying lengths of time, and/or retraction of the published work.

Name: Shoichi Maruyama
Manuscript ID: K360-2024-000725R1
Manuscript Title: "Plasma metabolite profiles between in-center daytime extended-hours and conventional hemodialysis
Date of Completion: November 5, 2024
Disclosure Updated Date: November 5, 2024

ASN
American Society of Nephrology
JASN
Journal of the American Society of Nephrology
KIDNEY360
Accessing Our World From Every Angle
CJASN
Clinical Journal of the American Society of Nephrology

ASN Journal Disclosure Form

As per ASN journal policy, I have disclosed any financial relationships or commitments I have held in the past 36 months as included below. I have listed my Current Employer below to indicate there is a relationship requiring disclosure. If no relationship exists, my Current Employer is not listed.

N. Nishibori reports the following:
Employer: Nagoya University

I understand that the information above will be published within the journal article, if accepted, and that failure to comply and/or to accurately and completely report the potential financial conflicts of interest could lead to the following: 1) Prior to publication, article rejection, or 2) Post-publication, sanctions ranging from, but not limited to, issuing a correction, reporting the inaccurate information to the authors' institution, banning authors from submitting work to ASN journals for varying lengths of time, and/or retraction of the published work.

Name: Nobuhiro Nishibori
Manuscript ID: K360-2024-000725R1
Manuscript Title: Plasma metabolite profiles between in-center daytime extended-hours and conventional hemodialysis
Date of Completion: November 7, 2024
Disclosure Updated Date: November 7, 2024

ASN
American Society of Nephrology
JASN
Journal of the American Society of Nephrology
KIDNEY360
Accessing Our World From Every Angle
CJASN
Clinical Journal of the American Society of Nephrology

ASN Journal Disclosure Form

As per ASN journal policy, I have disclosed any financial relationships or commitments I have held in the past 36 months as included below. I have listed my Current Employer below to indicate there is a relationship requiring disclosure. If no relationship exists, my Current Employer is not listed.

M. Okazaki reports the following:
Research Funding: Japan Society for the Promotion of Science Grants-in-Aid for Scientific Research; KAKENHI;

I understand that the information above will be published within the journal article, if accepted, and that failure to comply and/or to accurately and completely report the potential financial conflicts of interest could lead to the following: 1) Prior to publication, article rejection, or 2) Post-publication, sanctions ranging from, but not limited to, issuing a correction, reporting the inaccurate information to the authors' institution, banning authors from submitting work to ASN journals for varying lengths of time, and/or retraction of the published work.

Name: Masaki Okazaki
Manuscript ID: K360-2024-000725R1
Manuscript Title: Plasma metabolite profiles between in-center daytime extended-hours and conventional hemodialysis,
Date of Completion: November 5, 2024
Disclosure Updated Date: November 5, 2024

ASN American Society of Nephrology | JASN Journal of the American Society of Nephrology | KIDNEY360 Accessing Our World From Every Angle | CJASN Clinical Journal of the American Society of Nephrology

ASN Journal Disclosure Form

As per ASN journal policy, I have disclosed any financial relationships or commitments I have held in the past 36 months as included below. I have listed my Current Employer below to indicate there is a relationship requiring disclosure. If no relationship exists, my Current Employer is not listed.

T. Ozeki reports the following:
Employer: Nagoya University

I understand that the information above will be published within the journal article, if accepted, and that failure to comply and/or to accurately and completely report the potential financial conflicts of interest could lead to the following: 1) Prior to publication, article rejection, or 2) Post-publication, sanctions ranging from, but not limited to, issuing a correction, reporting the inaccurate information to the authors' institution, banning authors from submitting work to ASN journals for varying lengths of time, and/or retraction of the published work.

Name: Takaya Ozeki
Manuscript ID: K360-2024-000725R1
Manuscript Title: Plasma metabolite profiles between in-center daytime extended-hours and conventional hemodialysis
Date of Completion: November 7, 2024
Disclosure Updated Date: May 5, 2024

ASN Journal Disclosure Form

As per ASN journal policy, I have disclosed any financial relationships or commitments I have held in the past 36 months as included below. I have listed my Current Employer below to indicate there is a relationship requiring disclosure. If no relationship exists, my Current Employer is not listed.

R. Saito reports the following:
Employer: Institute for Advanced Biosciences, Keio University

I understand that the information above will be published within the journal article, if accepted, and that failure to comply and/or to accurately and completely report the potential financial conflicts of interest could lead to the following: 1) Prior to publication, article rejection, or 2) Post-publication, sanctions ranging from, but not limited to, issuing a correction, reporting the inaccurate information to the authors' institution, banning authors from submitting work to ASN journals for varying lengths of time, and/or retraction of the published work.

Name: Rintaro Saito
Manuscript ID: K360-2024-000725R1
Manuscript Title: Plasma metabolite profiles between in-center daytime extended-hours and conventional hemodialysis
Date of Completion: November 5, 2024
Disclosure Updated Date: November 5, 2024

ASN Journal Disclosure Form

As per ASN journal policy, I have disclosed any financial relationships or commitments I have held in the past 36 months as included below. I have listed my Current Employer below to indicate there is a relationship requiring disclosure. If no relationship exists, my Current Employer is not listed.

N. Takami has nothing to disclose.

I understand that the information above will be published within the journal article, if accepted, and that failure to comply and/or to accurately and completely report the potential financial conflicts of interest could lead to the following: 1) Prior to publication, article rejection, or 2) Post-publication, sanctions ranging from, but not limited to, issuing a correction, reporting the inaccurate information to the authors' institution, banning authors from submitting work to ASN journals for varying lengths of time, and/or retraction of the published work.

Name: Norito Takami
Manuscript ID: K360-2024-000725R1
Manuscript Title: Plasma metabolite profiles between in-center daytime extended-hours and conventional hemodialysis
Date of Completion: November 4, 2024
Disclosure Updated Date: November 4, 2024
