# Supplementary material for: Plasma Metabolite Profiles Between In-Center Daytime Extended-Hours and Conventional Hemodialysis
Source: Kidney360. 2024 Dec 9;6(3):420–31. doi: 10.34067/KID.0000000675 (PMC11970860; doi:10.34067/KID.0000000675)
Supplement: SUPPLEMENTARY MATERIAL [file kidney360-6-420-s002.pdf]

Supplemental Materials

Plasma metabolite profiles between in-center daytime extended-hours and conventional hemodialysis

Authors:

Norito Takami, Masaki Okazaki, Takaya Ozeki, Takahiro Imaizumi, Nobuhiro Nishibori, Shimon Kurasawa, Manabu Hishida, Shin'ichi Akiyama, Rintaro Saito, Akiyoshi Hirayama, Hirotake Kasuga, Fumika Kaneda, Shoichi Maruyama

Contents:

Supplemental Figure 1. Comparison of metabolite profiles on different dialysis methods by principal component analysis (PCA)

Supplemental Figure 2. Kaplan–Meier analysis of all-cause mortality of patients on extended-hours and those on conventional hemodialysis

Supplemental Table 1. The nutrition content of meals provided during extended-hours hemodialysis

Supplemental Table 2. A list of 117 metabolites included in the statistical analyses

Supplemental Table 3. Comparison of lactate-to-pyruvate ratio across different dialysis methods

Supplemental Table 4. Fitting of inverse probability of treatment-weighted propensity score

Supplemental Method 1. A brief description of the sample preparation and measurement process

Supplemental Figure 1. Comparison of plasma metabolic profiles on different dialysis methods by principal component analysis (PCA)

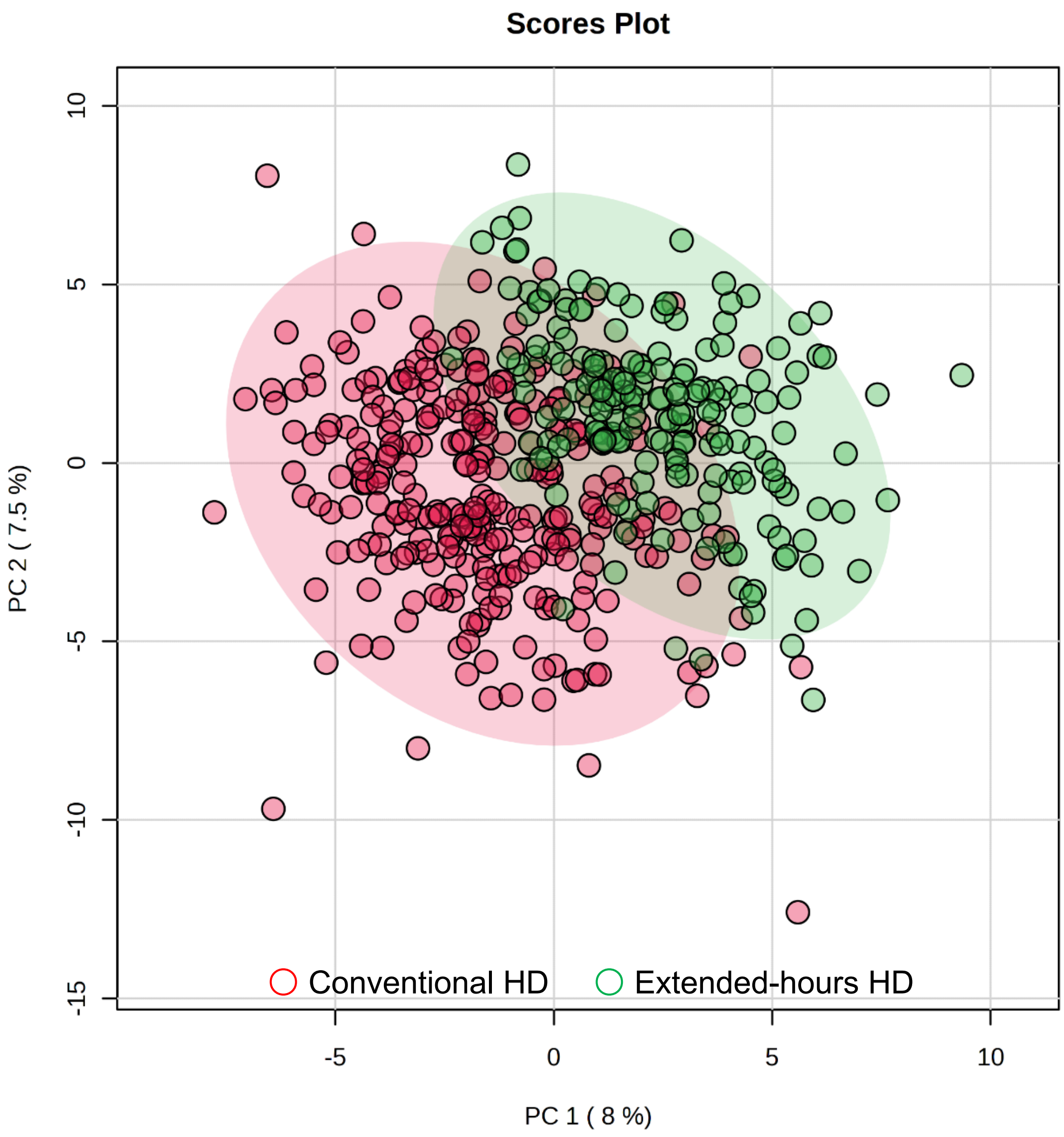

PCA score plot. Participants were grouped according to extended-hours (green) and conventional (red) hemodialysis. The score plot displays the separation between the two groups. Colored ellipses illustrate the 95% confidence intervals. Colored dots represent individual samples. Abbreviations: PC, principal component; HD, hemodialysis.

Supplemental Figure 2. Kaplan–Meier analysis of all-cause mortality of patients on extended-hours and those on conventional hemodialysis

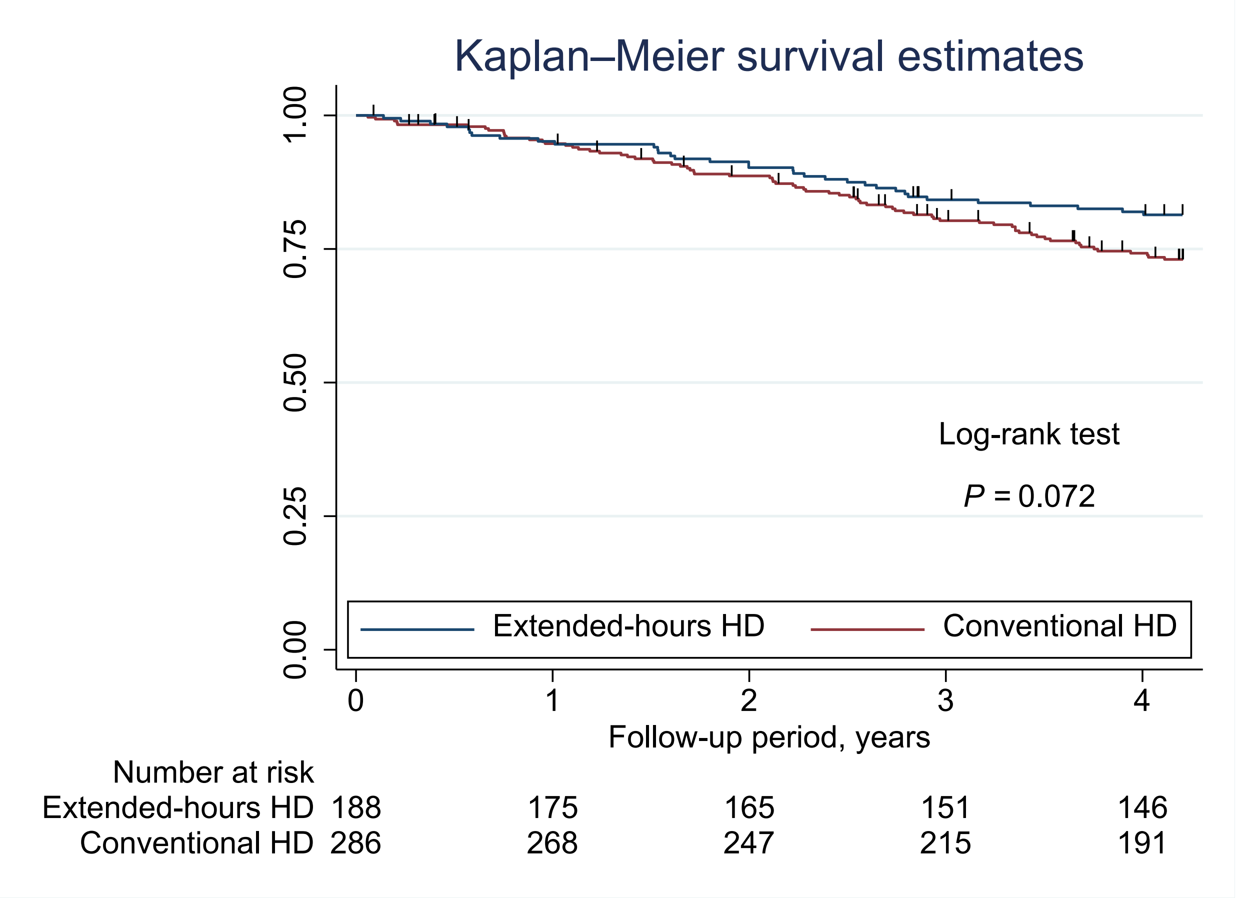

Abbreviation: HD, hemodialysis.

Supplemental Table 1. The nutrition content of meals provided during extended-hours hemodialysis

Information on intra-dialytic meal consumption	
Average nutrient value per serving	
Energy, kcal	741
Protein, g	30
Phosphorus, mg	383

The values represent the average nutritional content of meals provided during extended-hours hemodialysis sessions, calculated from the monthly meal plan.

Supplemental Table 2. A list of 117 metabolites included in the statistical analyses

Metabolites	KEGG ID or PubChem CID	Detection rate (extended-hours HD, conventional HD)
2,3-Pyridinedicarboxylate	C03722	100%, 100%
2-Aminobutanoate	C02356	100%, 100%
2-Hydroxybutyrate	C05984	100%, 100%
2-Hydroxyglutarate	C02630	72.3%, 86.4%
2-Hydroxyisobutyrate	C21297	91.5%, 95.1%
2-Hydroxypentanoate	PubChem CID: 13879500	100%, 100%
2-Isopropylmalate	C02504	96.3%, 93.4%
2-Oxoisopentanoate	C00141	95.7%, 97.9%
3-Aminoisobutyrate	C05145	100%, 100%
3-Hydroxy-3-methylglutarate	C03761	93.6%, 97.6%
3-Hydroxybutyrate	C01089	100%, 100%
3-Methylhistidine	C01152	100%, 100%
3-Phosphoglycerate	C00197	25.5%, 90.2%
4-Hydroxy-3-methoxymandelate	C05584	70.7%, 85.3%
4-Pyridoxate	C00847	100%, 100%
5-Oxohexanoate	C02129	69.1%, 98.6%
5-Oxoproline	C01879	100%, 100%
Asymmetric dimethylarginine	C03626	95.7%, 85.7%
Adenosine diphosphate	C00008	13.8%, 90.9%
Alanine	C00041	100%, 100%
Allantoate	C00499	75.5%, 78.7%
Arginine	C00062	100%, 100%
Argininosuccinate	C03406	73.9%, 52.8%
Asparagine	C00152	100%, 100%
Aspartic acid	C00049	100%, 100%
Azelate	C08261	100%, 100%
Betaine	C00719	100%, 100%
Biotin	C00120	75.0%, 73.1%
Carnitine	C00318	100%, 100%
Choline	C00114	100%, 100%
Cis-Aconitate	C00417	100%, 100%
Citraconate	C02226	96.8%, 96.5%
Citramalate	C02614	98.4%, 97.6%
Citrate	C00158	100%, 100%
Citrulline	C00327	100%, 100%
Creatine	C00300	100%, 100%
Creatinine	C00791	100%, 100%
Cystathionine	C02291	77.7%, 88.8%
Cysteine S-sulfate	C05824	90.4%, 100%
Cystine	C00491	100%, 100%
Decanoate	C01571	21.3%, 79.7%
Diethanolamine	C06772	100%, 97.2%

Ethanolamine phosphate	C00346	61.2%, 94.8%
Fumarate	C00122	94.1%, 98.6%
Glutamine	C00064	100%, 100%
Glutamic acid	C00025	100%, 100%
Glucuronate	C00191	100%, 100%
Glutarate	C00489	100%, 100%
Glycine	C00037	100%, 100%
Glycerophosphate	C00093	88.8%, 68.2%
Glycolate	C00160	93.1%, 96.9%
Guanidinoacetate	C00581	98.4%, 97.9%
Guanidinosuccinate	C03139	99.5%, 99.7%
Hippurate	C01586	100%, 100%
Histidine	C00135	100%, 100%
Homovanillate	C05582	77.7%, 83.6%
Hydroxyproline	C01157	100%, 100%
Hypotaurine	C00519	83.5%, 94.4%
Hypoxanthine	C00262	65.4%, 99.7%
Isoleucine	C00407	100%, 100%
Indole-3-acetate	C00954	98.4%, 97.6%
Indoxyl sulfate	PubChem CID: 10258	100%, 100%
Isethionate	C05123	100%, 100%
Isocitrate	C00311	100%, 100%
Kynurenine	C00328	100%, 100%
Lactate	C00186	100%, 100%
Leucine	C00123	100%, 100%
Lysine	C00047	100%, 100%
Malate	C00711	100%, 100%
Malonate	C00383	56.9%, 84.6%
Methionine	C00073	100%, 100%
Methionine sulfoxide	C02989	94.1%, 97.6%
Mucate	C00879	100%, 100%
N,N-Dimethylglycine	C01026	100%, 100%
N1-Acetylspermidine	C00612	91.0%, 75.5%
N6,N6,N6-Trimethyllysine	C03793	98.4%, 98.6%
N-Acetylaspartate	C01042	100%, 100%
N-Acetylglutamate	C00624	100%, 100%
N-Acetylneuraminate	C00270	100%, 100%
N-Epsilon-acetyllysine	C02727	56.4%, 78.0%
N-Formylmethionine	C03145	85.6%, 92.3%
O-Acetylcarnitine	C02571	100%, 100%
O-Hydroxyhippurate	C07588	75.5%, 68.5%
Ornithine	C00077	100%, 100%
Pelargonate	C01601	71.8%, 99.0%
Phenylalanine	C00079	100%, 100%
Phthalate	C01606	29.8%, 90.9%

Pimelate	C02656	99.5%, 99.7%
Pipecolate	C00408	100%, 100%
Proline	C00148	100%, 100%
Proline betaine	C10172	100%, 99.7%
Pyruvate	C00022	100%, 100%
Quinate	C00296	94.1%, 97.6%
Saccharate	C00818	100%, 100%
Sarcosine	C00213	100%, 100%
Symmetric dimethylarginine	PubChem CID: 169148	95.7%, 98.3%
Sebacate	C08277	43.6%, 73.8%
Serine	C00065	100%, 100%
Succinate	C00042	100%, 100%
Taurine	C00245	100%, 100%
Terephthalate	C06337	89.9%, 95.8%
Threonine	C00188	100%, 100%
Threonate	C01620	100%, 100%
trans-Aconitate	C02341	100%, 100%
Trigonelline	C01004	100%, 99.7%
Trimethylamine N-oxide	C01104	100%, 100%
Tryptophan	C00078	100%, 100%
Tryptophol	C00955	22.3%, 79.4%
Tyrosine	C00082	100%, 100%
Urate	C00366	100%, 100%
Urea	C00086	100%, 100%
Uridine	C00299	100%, 100%
Valine	C00183	100%, 100%
α-Aminoadipate	C00956	82.4%, 71.7%
α-Methylserine	C02115	72.9%, 80.1%
β-Alanine	C00099	100%, 100%
γ-Butyrobetaine	C01181	100%, 99.7%

Abbreviation: HD, hemodialysis.

Supplemental Table 3. Comparison of lactate-to-pyruvate ratio across different dialysis methods

	Extended-hours HD	Conventional HD	*P* value	Difference (Extended-hours HD vs. conventional HD)	Adjusted *P*
Lactate-to-pyruvate ratio	10.2 [8.9–11.6]	16.7 [14.3–19.6]	<0.001	–8.6 (–9.8 to –7.4)	<0.001

Values are presented as medians [interquartile ranges] or differences (95% confidence intervals). *P* values were derived from the Wilcoxson rank-sum test, and adjusted *P* was derived from propensity score-adjusted multiple regression analysis. Propensity scores were estimated using the following covariates: age, sex, total duration on dialysis, body mass index, diabetes mellitus, hypertension, coronary artery disease, peripheral artery disease, and cerebrovascular disease. Multiple regression analysis was adjusted for propensity scores, propensity score model variables, plus medication data (i.e., use of phosphate binders, active vitamin D, and calcimimetics). $P < 0.05$ was considered significant.

Abbreviations: HD, hemodialysis.

Supplemental Table 4. Fitting of inverse probability of treatment-weighted propensity score

	Mean in extended-hours HD	Mean in conventional HD	Abs. std. diff
Age, years	66.9	67.6	0.059
Sex	0.69	0.68	0.014
Duration on dialysis, months	119.8	116.1	0.035
Body mass index, kg/m^2	22.9	22.6	0.064
Diabetes mellitus	0.47	0.49	0.040
Hypertension	0.69	0.72	0.075
Coronary artery disease	0.28	0.28	0.010
Peripheral artery disease	0.20	0.23	0.075
Cerebrovascular disease	0.21	0.20	0.024

Categorical variables indicate proportion.
Abbreviations: HD, hemodialysis; Abs. std. diff, absolute standardized difference.

Supplemental Method 1. A brief description of the sample preparation and measurement process

Plasma (50 µL) was added to a methanol (MeOH) solution (450 µL) containing internal standards (20 µL each of methionine sulfone and D-camphol-10-sulfonic acid) and suspended. Chloroform (500 µL) and Milli-Q water (200 µL) were added to this solution, stirred, and centrifuged at 10,000 *g* for 3 min at 4 °C. In total, A 300 µL sample from the upper aqueous-MeOH layer was collected and subjected to ultrafiltration using a filter with a molecular weight cutoff of 5,000 Da, followed by centrifugal filtrate at 9,100 *g* for 120 min at 20°C. Finally, the filtrate was concentrated by centrifugation and used as the sample. Before measurement, the samples were dissolved in 50 µL of Milli-Q water containing internal standards 200 µM each of 3-aminopyrrolidine and trimesate and measured under CE-TOFMS conditions.
